# Supplementary material for: Microbial recognition by GEF-H1 controls IKKε mediated activation of IRF5
Source: Nat Commun. 2019 Mar 22;10:1349. doi: 10.1038/s41467-019-09283-x (PMC6430831; doi:10.1038/s41467-019-09283-x)
Supplement: Supplementary file 1 — Supplementary Information [file 41467_2019_9283_MOESM1_ESM.pdf]

Supplementary Information

Microbial Recognition by GEF-H1 Controls IKKε Mediated Activation of IRF5

Yun Zhao et al.

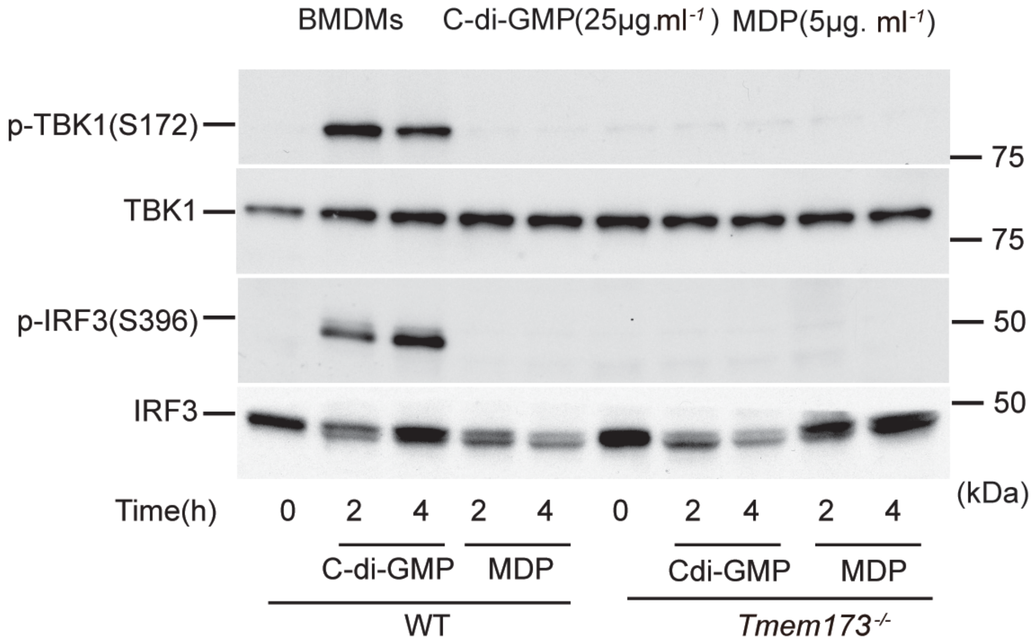

Supplementary Figure 1: MDP cannot induce TBK1 and IRF3 phosphorylation in macrophages.

Related to Figure 1

WT or STING deficient bone-marrow derived macrophages were stimulated with either C-di-GMP or N-glycolyl-MDP for the indicated time period and phosphorylation and expression of IRF3 and TBK1 determined by Western blotting.

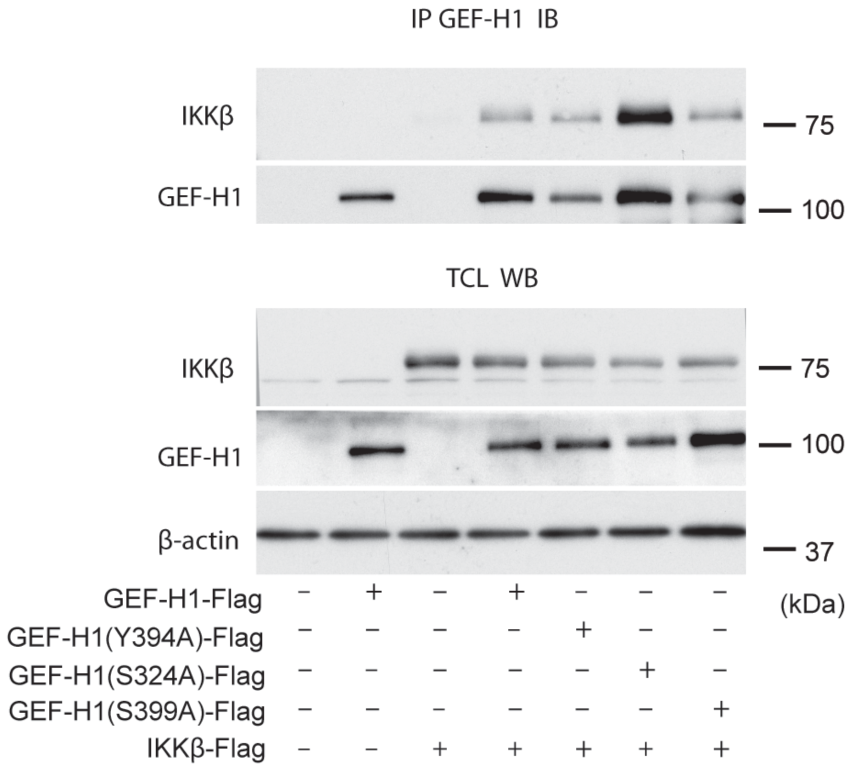

Supplementary Figure 2: GEF-H1 interacts with IKKβ

Related to Figure 3

WT or GEF-H1 variants and IKKβ were co-expressed in HEK293T cells, GEF-H1 pulled down with anti GEF-H1 and co-immunoprecipitated IKKβ detected by Western blotting.

Supplementary Table 1:

Primers used in this study

Genes	Forward 5’ to 3’	Reverse 5’ to 3’
18S	GTAACCCGTTGAACCCCATT	CCATCCAATCGGTAGTAGCG
Pglyrp1	TGTGCAAAGCACTCTCTCTC	TCACTAGGTCTCAAGGTCTCTC
GzmD	TGATTCTCCTGACCCTACTTCT	CAGGAAGCCTCCACAGTATATC
GzmE	GAGATACTGTGGAGGCTTCTTG	CCTCCTTAGCCTTGATGTTGT
Serpine1	GAGGTGGAAAGAGCCAGATTTA	CCACTGAAGTAGAGGGCATTC
NOD2	AGCCCTGGCTGAAGTTGTAG	TTGAGTCCTTCTGCGAGAGA
