# Supplementary material for: Microbial recognition by GEF-H1 controls IKKε mediated activation of IRF5
Source: Nat Commun. 2019 Mar 22;10:1349. doi: 10.1038/s41467-019-09283-x (PMC6430831; doi:10.1038/s41467-019-09283-x)
Supplement: Supplementary file 3 — Description of Additional Supplementary Files [file 41467_2019_9283_MOESM3_ESM.pdf]

Description of Additional Supplementary Files

Supplementary Data 1

Related to Figure 5, Gene clusters that were either dependent on GEF-H1 and IRF5, GEF-H1 or IRF5 alone or that were induced by MDP independently of GEF-H1 and IRF5.
