# Supplementary material for: Microbial recognition by GEF-H1 controls IKKε mediated activation of IRF5
Source: Nat Commun. 2019 Mar 22;10:1349. doi: 10.1038/s41467-019-09283-x (PMC6430831; doi:10.1038/s41467-019-09283-x)
Supplement: Supplementary file 5 — Reporting Summary [file 41467_2019_9283_MOESM5_ESM.pdf]

nature research

Corresponding author(s): NCOMMS-18-21793B

Last updated by author(s): Feb 15, 2019

Reporting Summary

Nature Research wishes to improve the reproducibility of the work that we publish. This form provides structure for consistency and transparency in reporting. For further information on Nature Research policies, see Authors & Referees and the Editorial Policy Checklist.

Statistics

For all statistical analyses, confirm that the following items are present in the figure legend, table legend, main text, or Methods section.

n/a	Confirmed	
☐	☒	The exact sample size (n) for each experimental group/condition, given as a discrete number and unit of measurement
☐	☒	A statement on whether measurements were taken from distinct samples or whether the same sample was measured repeatedly
☐	☒	The statistical test(s) used AND whether they are one- or two-sided<br>*Only common tests should be described solely by name; describe more complex techniques in the Methods section.*
☐	☒	A description of all covariates tested
☒	☐	A description of any assumptions or corrections, such as tests of normality and adjustment for multiple comparisons
☐	☒	A full description of the statistical parameters including central tendency (e.g. means) or other basic estimates (e.g. regression coefficient) AND variation (e.g. standard deviation) or associated estimates of uncertainty (e.g. confidence intervals)
☐	☒	For null hypothesis testing, the test statistic (e.g. F, t, r) with confidence intervals, effect sizes, degrees of freedom and P value noted<br>*Give P values as exact values whenever suitable.*
☒	☐	For Bayesian analysis, information on the choice of priors and Markov chain Monte Carlo settings
☒	☐	For hierarchical and complex designs, identification of the appropriate level for tests and full reporting of outcomes
☒	☐	Estimates of effect sizes (e.g. Cohen's d, Pearson's r), indicating how they were calculated

Our web collection on statistics for biologists contains articles on many of the points above.

Software and code

Policy information about availability of computer code

Data collection	Volocity (Perkin Elmer) Image acquisition, CFX Manager Software for qPCR
Data analysis	Tophat v1.2.5, Cuffdiff v1.06, Seqmonk v1.0, PRIZM, Excel, Volocity, ImageJ

For manuscripts utilizing custom algorithms or software that are central to the research but not yet described in published literature, software must be made available to editors/reviewers. We strongly encourage code deposition in a community repository (e.g. GitHub). See the Nature Research guidelines for submitting code & software for further information.

Data

Policy information about availability of data

All manuscripts must include a data availability statement. This statement should provide the following information, where applicable:

- Accession codes, unique identifiers, or web links for publicly available datasets
- A list of figures that have associated raw data
- A description of any restrictions on data availability

RNA sequencing data that support the findings of this study have been deposited in GEO with the accession codes GSE126749. http://www.ncbi.nlm.nih.gov/geo/query/acc.cgi?acc=GSE126749.

The authors declare that all other data supporting the findings of this study are available within the paper and its supplementary information.

Field-specific reporting

Please select the one below that is the best fit for your research. If you are not sure, read the appropriate sections before making your selection.

☒ Life sciences ☐ Behavioural & social sciences ☐ Ecological, evolutionary & environmental sciences

For a reference copy of the document with all sections, see nature.com/documents/nr-reporting-summary-flat.pdf

Life sciences study design

All studies must disclose on these points even when the disclosure is negative.

Sample size	Sample size were determined based on the number of BM derived macrophages that can be isolated from mice.
Data exclusions	Every experiment was repeated at least three times. No data was excluded.
Replication	All experiments were successfully replicated multiple times
Randomization	Not specific selection was carried for experimental animals.
Blinding	Analysis of nuclear translocation of IRF5 was carried in a blinded fashion.

Reporting for specific materials, systems and methods

We require information from authors about some types of materials, experimental systems and methods used in many studies. Here, indicate whether each material, system or method listed is relevant to your study. If you are not sure if a list item applies to your research, read the appropriate section before selecting a response.

Materials & experimental systems

n/a	Involved in the study	
☐	☒	Antibodies
☐	☒	Eukaryotic cell lines
☒	☐	Palaeontology
☐	☒	Animals and other organisms
☒	☐	Human research participants
☒	☐	Clinical data

Methods

n/a	Involved in the study	
☒	☐	ChIP-seq
☒	☐	Flow cytometry
☒	☐	MRI-based neuroimaging

Antibodies

Antibodies used	antibodies against GEF-H1 (ab155785; 1/2000 dilution), phospho-GEF-H1 (S885, ab94348; 1/2000 dilution), IRF5 (ab21689; 1/1000 dilution), Phospho-ROCK1(T455 /S456, ab203273; 1/1000 dilution), ROCK1 (EP786Y, ab45171; 1/1000 dilution) were from Abcam. The antibody against phospho-Ser445 IRF5 used at 1/2000 dilution was generated by immunizing rabbits with a synthetic peptide (IRLQIpS445NPDLC) (NeoBiolab, MA. USA). Phospho-p65-NFκB (Ser536, 93H1; 1/1000 dilution), p65-NFκB (D14E12; 1/1000 dilution), phospho-IRF3 (Ser396, 4D4G; 1/1000 dilution), IRF3 (D83B9; 1/1000 dilution), Phospho-IKKα/β (Ser176/180, 16A6; 1/1000 dilution), IKKβ (D30C6; 1/1000 dilution), Phospho-IKKε (Ser172, D1B7; 1/1000 dilution), IKKε (2690; 1/1000 dilution), β-actin (8H10D10; 1/10000 dilution), and anti-Lamin A/C (4C11; 1/1000 dilution) antibodies were purchased from Cell Signaling Technology. Anti-FLAG (F7425; 1/3000 dilution) and anti-HA (H9658; 1/3000 dilution) antibodies were obtained from Sigma.rabbit (NA934V, GE Healthcare) HRP were used at 1/5000 and incubated for 1h at RT. For IP we used true blot anti rabbit (ROCKLAND; 18-8816-33) or ULTRA anti mouse (ROCKLAND; 18-8817-33) HRP at 1/4000.
Validation	The antibody against phospho-Ser445 IRF5 was verified using IRF5 deficient mice. Other ABs were used according to manufactures recommendation and size of expected proteins verified.

Eukaryotic cell lines

Policy information about cell lines

Cell line source(s)	HEK293T and ARPE-19 cells were purchased from American Type Culture Collection.
Authentication	Cell lines are confirmed by the American Type Culture Collection.
Mycoplasma contamination	The cells were tested for mycoplasma by PCR and found to be negative.

Commonly misidentified lines
(See ICLAC register)

n/a

Animals and other organisms

Policy information about studies involving animals; ARRIVE guidelines recommended for reporting animal research

Laboratory animals	C57BL/6 WT (Wild-type),Irf5-/-, Nod2-/- and Ikkε-/- animals were obtained from Jackson Laboratory (Bar harbor, ME). GEFH1 deficient mice were generated in the Reinecker laboratory and gene targeting confirmed by PCR and RNAsequencing.
Wild animals	No wild animals were used
Field-collected samples	No field-collected samples were used
Ethics oversight	Protocols were approved by the subcommittee on Research Animal Care at the Massachusetts General Hospital.

Note that full information on the approval of the study protocol must also be provided in the manuscript.
